# *Xylosma* G. Forst. Genus: Medicinal and Veterinary Use, Phytochemical Composition, and Biological Activity

**DOI:** 10.3390/plants11091252

**Published:** 2022-05-05

**Authors:** Rodrigo Duarte-Casar, Juan Carlos Romero-Benavides

**Affiliations:** 1 Maestría en Química Aplicada, Facultad de Ciencias Exactas y Naturales, Universidad Técnica Particular de Loja, Loja 110108, Ecuador; rduarte@utpl.edu.ec; 2 Departamento de Química, Facultad de Ciencias Exactas y Naturales, Universidad Técnica Particular de Loja, Loja 110108, Ecuador

**Keywords:** *Xylosma*, ethnopharmacology, phytochemicals, *Salicaceae*, biological activity

## Abstract

*Xylosma* G. Forst. is a genus of plants belonging to the *Salicaceae* family with intertropical distribution in America, Asia, and Oceania. Of the 100 accepted species, 22 are under some level of conservation risk. In this review, around 13 species of the genus used as medicinal plants were found, mainly in Central and South America, with a variety of uses, among which antimicrobial is the most common. There is published research in chemistry and pharmacological activity on around 15 of the genus species, centering in their antibacterial and fungicidal activity. Additionally, a variety of active phytochemicals have been isolated, the most representative of which are atraric acid, xylosmine and its derivatives, and velutinic acid. There is still ample field for the validation and evaluation of the activity of *Xylosma* extracts, particularly in species not yet studied, and concerning uses other than antimicrobial and for the identification and evaluation of their active compounds.

## 1. Introduction

The use of medicinal plants is not exclusive to humans, but is also reported in superior apes and other animal species [1,2]; it is therefore not surprising that humans have used medicinal plants since the earliest antiquity [3,4,5]. Until recently, the approach was purely empirical [6], but today, this knowledge is being validated and refined by modern research methodology that accelerates the generation of knowledge and its applications [7]. Today, natural products are an important source of new drugs and treatments, either directly or through chemical modifications [8].

In this article we performed a systematic review of the phytochemical composition, pharmacological, medical, and veterinary applications on the species in the genus, gathering the existing information in scientific literature about the ethnomedical knowledge, the active molecules identified and isolated from them, and the research studies that validate their potential efficacy. The objective of the study is to identify gaps in the knowledge about the genus and find study lines that may guide future research.

## 2. Genus

The *Salicaceae* Mirb. family, to which the *Xylosma* genus belongs, is famously medicinal because of the *Salix* genus (willow), the pharmacological properties of which were already used in ancient Mesopotamia, and were extolled in the first century CE, in Dioscorides’ *De Materia Medica* [8,9].

The *Xylosma* genus is one of the 55 that conform the *Salicaceae* family [9], and is composed of 100 accepted species [10], although others list 45 [11]. Until recently, it was included in the now-deprecated *Flacourtiaceae* family, but has now been assigned to *Salicaceae* [12]. The name stems from the Greek words for “wood” and “smell” in reference to odoriferous quality of the wood of some Pacific species of the genus [11], presumably *X. orbiculata* and *X. suaveolens* used to perfume coconut oil by early South Pacific inhabitants [13]. At first, the genus was named *Myroxylon* (myrrh-wood) but was changed to *Xylosma* to avoid confusion with South American balsam trees [14]. Not all species in the genus are sweet-smelling: *X. maidenii* timber, for example, is foul-smelling. *Xylosma* species are described in detail by Woodson et al. [15].

In shrubs or small trees, often with axillary spines, the branchlets commonly lenticellate. Leaves alternate, sometimes borne in fascicles, usually short-petiolate, estipulate, the blade is often ±coriaceous, usually glandular-dentate, penninerved, rarely entire-margined, without pellucid-glands. Inflorescences axillary, fasciculate or contracted-racemose, and are rarely racemose. Flowers are small, dioecious, or rarely polygamous; pedicels are articulated above the base, and the bracts are minute; sepals 4-5(-6), imbricate, usually scale-like, slightly connate at the base, often ciliolate along the margins, usually persistent; petals none; stamens ∞ (8–35 in Panamanian spp.), usually surrounded by an annular or glandular, fleshy disc, the filaments free, filiform, short- to usually long-exserted, the anthers minute, basifixed, extrose, longitudinally dehiscent; ovary sessile, inserted on an annular disc, 1-locular, with 2–3, rarely 4–6, parietal placentas, each placenta with 2, sometimes 4–6, ovules, the style entire or ±divided, sometimes very short, the stigmas scarcely dilated to dilated; rudimentary ovary wanting in male flowers. Fruits baccate, rather dry, indehiscent, surmounted by the persistent style, the pericarp rather thin-coriaceous, the seeds 2–8, +angular by mutual pressure, the testa thin; endosperm copious; embryo large, the cotyledons broad.

Species in the *Xylosma* genus have several uses and properties, from landscaping (*Xylosma congesta* (Lour.) Merr.), beekeeping (*Xylosma venosa* N. E. Br. [16]), timber, firewood, to food and medicine; notably *Xylosma longifolia* Clos. Due to the thorns that some species of the genus have, common names such as “do not touch me” (*Xylosma coriacea* (Poit.) Eichler) or “deer antlers” (*Xylosma spiculifera* (Tul.) Triana and Planch.) are used for them [17]. Eleven species of the genus, particularly *Xylosma vincentii* Guillaumin, are known to be nickel hyperaccumulators [18,19] which presents potential for phytoremediation and phytomining [20].

## 3. Distribution and Localization

**Figure 1:**
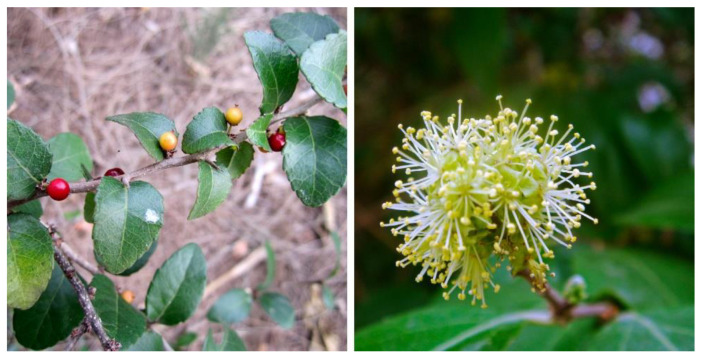
*Xylosma flexuosa* (Kunth) Hemsl. leaves and berries, left. *Xylosma congesta* (Lour.) Merr. inflorescence, right. Image sources: left, Public Domain (CC0); right, Miwasatosi, GDFL license.

**Figure 2:**
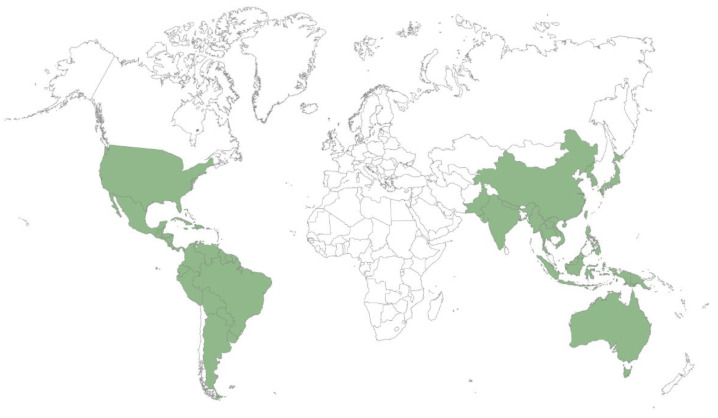
Worldwide *Xylosma* distribution, by country, after [10].

Species belonging to the *Xylosma* genus are present in subtropical America, Southeast Asia, and Oceania. Of the 100 species listed in the genus [10], 61 are found in America, 8 in Asia, and 31 in Oceania. Figure 1 shows examples of species of the genus. The map in Figure 2 shows the intertropical, and to a lesser extent, temperate, distribution of *Xylosma* species, by country.

Of the 100 species of the genus, 7 are listed as vulnerable, 9 as endangered, and 6 as critically endangered. In total, 22% of the species in the genus are considered as species of concern [21]. This should be considered when evaluating potential industrial uses for these species.

## 4. Methodology

Published works—articles and patents—were searched in Dimensions [22] for bibliometric data, and in scientific databases—Science Direct, Google Scholar, and Scopus—both using a browser interface and through Harzing’s “Publish or Perish” software [23] for each species of the genus, using inverted commas for an exact match, e.g., “*Xylosma benthamii*”. Relevant articles were selected after removing search terms unrelated to the area of interest, such as reforestation or drought resistance. When abundant results were obtained, the search was refined with more specific terms, for example “*Xylosma longifolia* medicinal” or “*Xylosma longifolia* ethnopharmacology”. Duplicate articles were removed, and the remaining articles were reviewed with focus in ethnopharmacological uses, phytochemical composition, and biological activity. When possible, the latest articles—no older than 10 years—have been cited. Preprints were not included. Due to the scarcity of sources, gray documentation such as books and thesis dissertations were included when they provided information not available in other sources.

**Figure 3:**
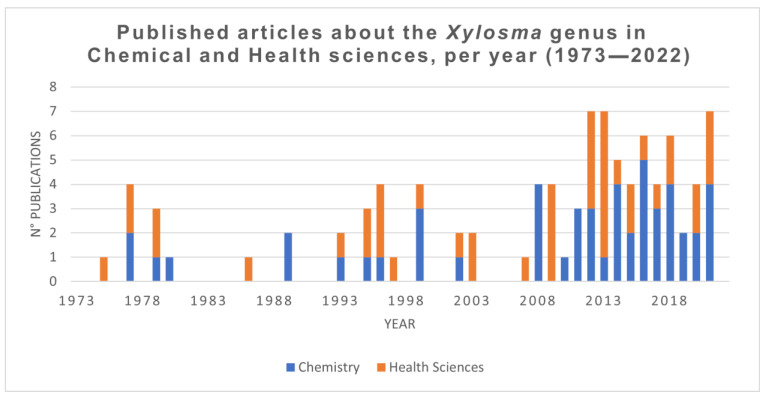
Publications containing the word *Xylosma* since the year 1973 in Medical and Health sciences, and in Chemistry. Data source: [22].

The research interest in *Xylosma* species in medical and health sciences has increased slowly during the last fifty years. Figure 3 shows the number of publications that include the word *Xylosma* in the document text in the fields mentioned. Even though the genus shows low research interest, a steady increase in appearances can be seen, with the last decade garnering much of the publication volume.

Genus *Xylosma* shares several secondary metabolite compounds and structures with *Flacourtia*. Both were recently reassigned into the *Salicaceae* family from *Flacourtiaceae*. Indeed, they share genetical characters between them and with other genera from *Salicaceae*, such as *Scolopia*, *Dovyalis*, and *Oncoba* [24].

## 5. Ethnopharmacological and Ethnoveterinary Usage

Of the 100 species of the genus, few appear in the scientific literature, and even fewer are mentioned from an ethnopharmacological or ethnoveterinary perspective. Notwithstanding, *Xylosma* species are a part of the traditional Chinese medicinal system, with documented uses of *X. congesta* appearing as early as the XVI century CE [25].

Few of the *Xylosma* species are recognized as medicinal. Table 1 summarizes the species with reported medicinal use along with their stated ethnopharmacological uses, when available. The Anatomical Therapeutic Chemical (ATC) Classification by the World Health Organization (WHO) is used to classify the uses for each species [26]. Not all species are identified in the literature, with general mentions as “*Xylosma* sp.” in some cases.

**Figure 4:**
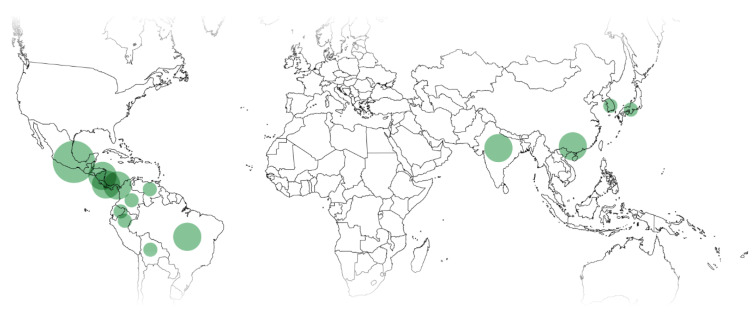
Ethnopharmacological and ethnoveterinary uses of *Xylosma* spp. Circle diameter proportional to use reports for the country.

Most *Xylosma* species in use are from Central and South America (38% and 31%), followed by China (23%) and India (8%). This is roughly in accordance with the local abundance of species. There are no reports of ethnomedicinal uses of *Xylosma* in Oceania. Uses by country are shown in Figure 4.

According to the ATC classification, the most frequent uses of *Xylosma* spp. in ethnopharmacology are dermatological, nervous system, and respiratory system, with 17% of the uses each, alimentary tract and metabolism with 11%, and genitourinary system and sex hormones with 6%. Additionally, 11% of the uses are veterinary.

As to the morphological structures used, the most common are leaves and barks with 33% each, and both stems and roots with 11% each.

## 6. Biological Activity

Biological activity tests of *Xylosma* have been carried out mostly in vitro, with no reported in vivo research, with plant extracts, be they leaf, root, bark, or the whole plant. Different solvents and solvent mixtures have been used for the extracts, mainly methanol and ethanol.

### In Vitro Activity

**Figure 5:**
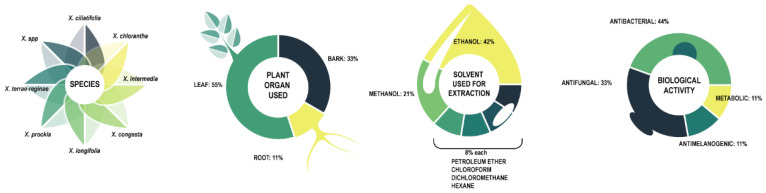
Summary of in vitro activity of *Xylosma* species.

In vitro research on biological activity of *Xylosma* species centers around 7 identified species and one unspecified one. The research figures are summarized in Figure 5, and the research is detailed in Table 2.

In vitro biological activity tests devote the most attention to leaves (55%), with bark (33%) and root (11%) used to a lesser extent. Extraction solvents are ethanol (42%), methanol (21%), and to a lesser extent petroleum ether, chloroform, dichloromethane, and hexane, with 8% each. The solvent choices support the assumption that most active compounds are polar, and are thus extracted with polar solvents.

Testing centers on antibacterial (44%) and antifungal (44%) activity reflects the main ethnopharmacological use but appears to leave other traditional uses unexplored.

Cytotoxicity assays involving *Xylosma* extracts show no significant cytotoxicity for *Xylosma prockia* nor for *Xylosma congesta* leaf extracts [52,55]. Moderate cytotoxicity was reported for methanol *Xylosma terrae reginae* extracts [53]. 2,6-dimethoxybenzoquinone (**33**) isolated from *Xylosma velutina* is reported as cytotoxic [56].

Even though there is no in vivo research concerning *Xylosma* species in the literature, there are several patents that include *Xylosma* extracts for cosmetic, veterinary, and traditional medicinal uses, such as hangover cures [33].

## 7. Phytochemical Composition

Phytochemical studies allow for the identification, separation, and isolation of compounds of interest [57]. Based on phytochemical screenings and other results published in the literature, the most common metabolites are alkaloids, terpenoids, and phenolics, among which flavonoids and the distinctive, often glycosylated, dihydroxyphenyl alcohol derivatives (xylosmin, xylosmacin etc.) abound [58,59]. These are also abundant in *Flacourtia (Salicaceae)* spp extracts, and several flacourtins have been isolated [60], which have shown antimalarial [61] and antiviral [62] activity.

**Figure 6:**
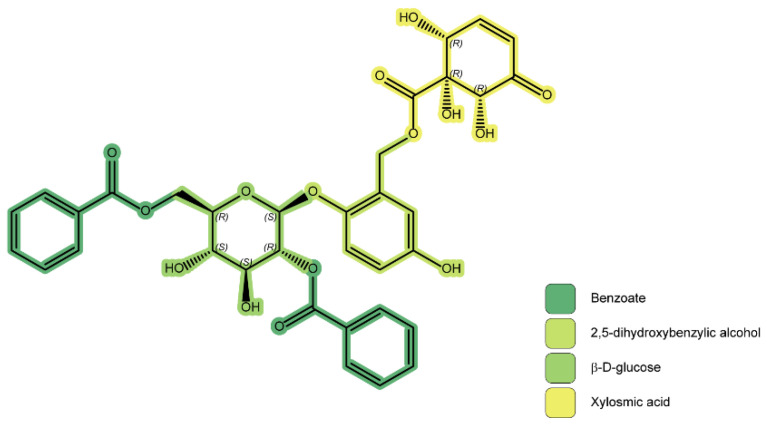
Xylosmin (**1**) structure. Moieties are highlighted as follows: xylosmic acid in yellow, benzoates in teal, d-β-glucose in light green, 2,5-dihydroxybenzylic alcohol in yellow-green.

First isolated from the Central and South American *Xylosma velutina* (Tul.) Triana and Planch and considered an “iconic compound” of the genus [63], xylosmin (**1**) is composed of a glucose unit, two esterified benzoic acid units, a 2,5-dixydroxybenzilic alcohol, and a (1*R*,2*R*,6*R*)-1,2,6-trihydroxy-5-oxocyclohex-3-ene-1-carboxylic acid, often named “xylosmic acid”. Figure 6 shows the structure of **1**, with the units highlighted in color.

After the isolation and identification of **1**, several related compounds from *Xylosma* and *Flacourtia* genera, among others, have been isolated, some of which have been found to present antiplasmodial and antiviral activity [61,64]. Xylosmin also exhibits phosphodiesterase inhibitory activity [65] which could explain the use of a non-specified *Xylosma* sp. against spider bites [48].

Xylocosides are phenylpropanoid compounds and phenolic glycosides isolated from the Asian *Xylosma controversa* Clos [35]. Xylocoside G (**11**) shows neuroprotective effect against β-amyloid neurotoxicity [66].

Atraric acid or methyl 2,4-dihydroxy-3,6-dimethylbenzoate (**16**) was isolated from *Xylosma velutina* [67] and presents antifungal [40,68], anti-inflammatory [69], and antiandrogenic activity [70], which has led to the patenting of the acid and its alkylated derivatives in the treatment of prostate hyperplasia, carcinoma, and spinobulbar muscular atrophy [71].

Some compounds found in plants belonging to the *Xylosma* genus, classified according to their chemical structure, are listed in Table 3. Where applicable, the biological activity of the identified compound has been mentioned.

Compounds have been isolated almost exclusively using chromatographic techniques, and have been identified through spectroscopical and spectrometric methods and by comparison with existing samples and published data [57].

**Figure 7:**
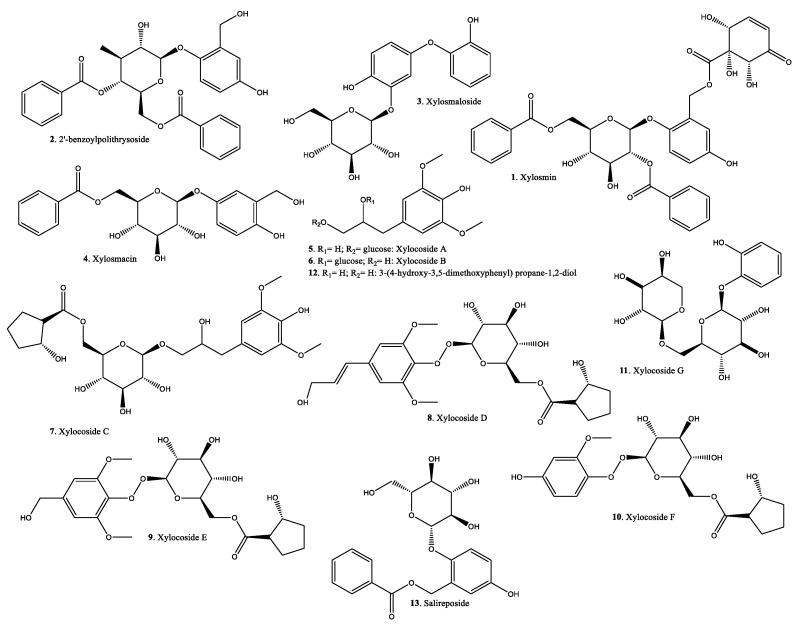
Characteristic compounds identified in *Xylosma* extracts.

**Figure 8:**
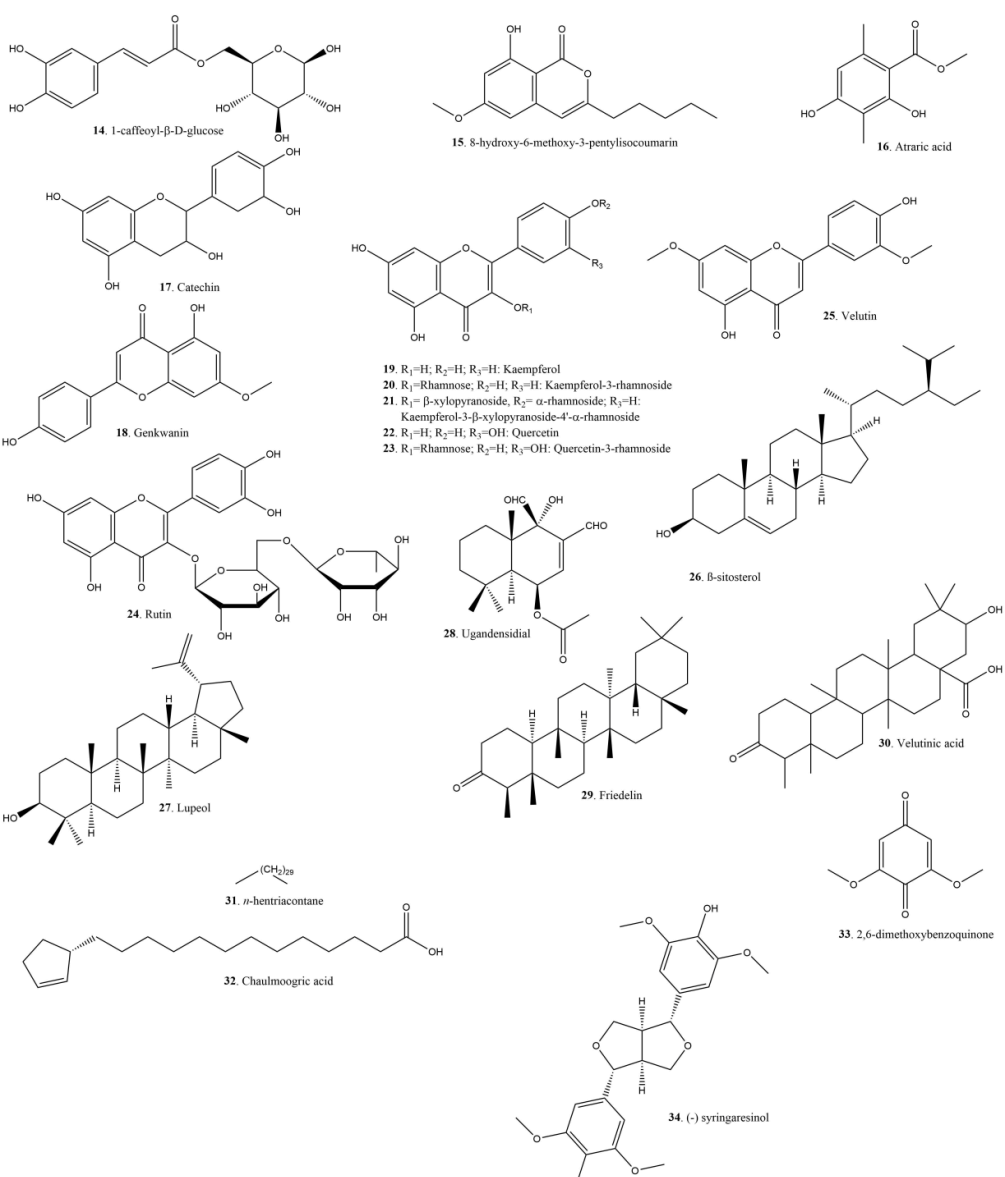
Flavonoids, terpenoids, and other compounds identified in *Xylosma* extracts.

Figure 7 and Figure 8 show the structure of some of the compounds identified in *Xylosma* spp. extracts. As expected in plant extracts, there is a variety of secondary metabolites in the form of terpenoids and flavonoids. There is a series of less usual phenolic compounds in the shape of dihydroxybenzyl alcohols and their glycosylated derivatives, esters, and ethers.

A strength of the genus is the potential for research it still holds: not many of its species have been systematically analyzed and interesting bioactivity can be found in previously unresearched species, as is the case with *X. prockia* [52]. A weakness is the conservation threat several of its species are under, particularly those from Oceania.

## 8. Conclusions

Species belonging to the *Xylosma* genus have several uses as food, medicine, wood, bird and pollinator attractors, etc. Among the medical uses, the ongoing research centers around the antibacterial, antifungal, anti-melanogenic, and antioxidant activity of *Xylosma* extracts, and other ethnopharmacological uses appear to have received less attention. This is seen as an opportunity for further study. 

Several bioactive compounds have been isolated from *Xylosma* species, some of which have pharmacological potential, such as atraric acid, used in cancer treatments.

There are several species of the genus—more than 80%—that have not been systematically studied, especially in America. This presents a research opportunity.

## Figures and Tables

**Table 1:** Medicinal and veterinary use of *Xylosma* species, listed in alphabetical order.

No.	Species	Region	Plant Organs Used	Use	Form of Usage	ATC Category	Ref.
**1**	*Xylosma benthamii* (Tul.) Triana and Planch.	Brazil	NS	Medicinal (not specified)	NS	NS	[27]
**2**	*Xylosma characantha* Standl.	Nicaragua	Leaves	Placentary retention in cattle	Decoction	Vet.	[28]
**3**	*Xylosma chlorantha* Donn. Sm.	Costa Rica	Bark	Medicinal (not specified)	NS	NS	[29]
**4**	*Xylosma ciliatifolia* (Clos) Eichler	Brazil	Root bark	Antibacterial	NS	V	[30]
**5**	*Xylosma congesta* (Lour.) Merr.	China Japan Korea	Bark Leaves	NS Anti-inflammatory Disease prevention in suckling piglets Birthing aid	Bark ashes Poultice	NS D G Vet.	[31] [32] [33] [34]
**6**	*Xylosma controversa* Clos.	Guangxi, China	Roots Leaves	NS	NS	NS	[35]
**7**	*Xylosma flexuosa* (Kunth) Hemsl.	Mexico	NS	Antipyretic Anti-tuberculosis	NS	N R	[36,37]
**8**	*Xylosma horrida* Rose.	Mexico Nicaragua Costa Rica	Bark	Kidneys	Decoction	G	[38]
**9**	*Xylosma intermedia* (Seem.) Triana and Planch.	Bolivia	Bark	Toothache	NS	N	[39]
**10**	*Xylosma longifolia* Clos	India China	Leaves Stem bark	Antifungal Antispasmodic Antidiarrheic Anti-tuberculosis Muscle sprains Narcotic	Paste Decoction Extract	D A A R M N	[40] [41] [42] [43] [44]
**11**	*Xylosma panamensis* (Turcz)	Panama Mexico	Bark Leaves	Cough Bronchitis	Dried	R	[45]
**12**	*Xylosma spiculifera* (Tul.) Triana and Planch	Colombia, Venezuela	Leaves	Ulcers, Dermatitis	Decoction	D	[46]
**13**	*Xylosma tessmanii* Sleumer	Ecuador	Leaves	Medicinal (NS)	NS	NS	[47]
**14**	*Xylosma sp.* (not specified)	Panama	Stem Root	Spider bites	Infusion	V	[48]
**15**	*Xylosma sp.* (not specified)	Perú	Bark	Bronchitis (with other plant species)	Decoction	R	[49]

NS: Not specified. ATC categories are as follows. A: Alimentary tract and metabolism, B: Blood and blood forming organs, C: Cardiovascular system, D: Dermatological, G: Genito urinary system and sex hormones, H: Systemic hormonal preparations, excluding sex hormones and insulins, J: Anti-infective for systemic use, L: Antineoplastic and immunomodulating agents, M: Musculo-skeletal system, N: nervous system, P: Antiparasitic products, insecticides, and repellents, R: Respiratory system, S: Sensory organs; V: Various [26]; STDs: Sexually transmitted diseases, Vet: veterinary.

**Table 2:** In vitro activity of *Xylosma* extracts. Species are in alphabetical order.

Species	Extract	Plant Organs Used	Biological Activity	Biological Model	Effect	Methodology	Ref.
*X. ciliatifolia*	Ethanol/Hexane partition	Root bark	Antibacterial	*S. aureus* *S. epidermis* *S. typihimurium* *E. coli*	Effective against *S. aureus* *S. epidermis* MIC (µg/mL) 250, 500	Disk diffusion assay	[30]
*X. clorantha*	Ethanol	Leaves	Metabolic syndrome	HepG2 cells	LXR 2.14 ± 0.11: 100 µg/mL	LXR transcriptional activity	[50]
*X. congesta*	Ethanol	Leaves	Anti-melanogenic	B16F10 cells	Melanin synthesis inhibition: up to 57.9%	α-MSH	[32]
*X. intermedia*	DCM/Ethanol	Bark	Antibacterial	*Bacillus cereus* *S. aureus*	MIC (ppm) 156 512	Microbroth dilution	[51]
*X. longifolia*	Petroleum ether Chloroform Methanol	Leaves, Stem bark	Antifungal	*Microsporum boullardii*, *M. canis*, *M. gypseum* *Trichophyton ajelloi* *T. rubrum*	MIC (mg/mL) 0.141–9.0	Agar diffusion Micro wells diffusion	[40]
*X. prockia*	Ethanol	Leaves	Antifungal	*Cryptococcus* spp.	MIC (ppm) 8–64	Antifungal microdilution susceptibility standard test	[52]
*X. terrae reginae*	Methanol	Root	Antibacterial Antifungal	*S. aureus* *C. albicans*	MIC (mg/mL) 2.5 1.2	Dilution method	[53]
*X. sp II*	Methanol	Leaves	Antibacterial	*Flavobacterium columnae*	MIC 375 µg/mL	Agar diffusion assay	[54]

DCM: Dichloromethane; MIC: Minimum inhibitory concentration; α-MSH: melanocyte-stimulating hormone. LXR: LXRα Fold Activation.

**Table 3:** Compounds isolated/identified in *Xylosma* extracts and oils and their biological effect.

No.	Compound	Identified/Isolated	Species	Collection Area	Plant Organ Used	Use	Effect	Ref.
**1**	Xylosmin	Y/Y	*X. velutina* *X. flexuosa*	Colombia Guanacaste, Costa Rica	Aerial parts	Antiviral Anti venom	RNA polymerase inhibition PDE inhibition	[72] [73] [62] [65]
**2**	2′-benzoylpoliothrysoside	Y/Y	*X. flexuosa*	Guanacaste, Costa Rica	Aerial parts			[73]
**3**	Xylosmaloside	Y/Y	*X. longifolia*	North-east India	NS	Antioxidant		[42]
**4**	Xylosmacin	Y/Y	*X. velutina*	NS	Stem bark			[67]
**5** **6** **7** **8** **9** **10**	Xylocosides A-F	Y/Y	*X. controversa*	Guangxi, China	Stems			[35]
**11**	Xylocoside G	Y/Y	*X. controversa*	Guangxi, China	Stems		Neuroprotective	[35] [66]
**12**	3-(4-hydroxy-3,5-dimethoxyphenyl)propane-1,2-diol	Y/Y	*X. controversa*	Guangxi, China	Stems			[35]
**13**	Salireposide	Y/Y	*X. flexuosa*	Guanacaste, Costa Rica	Aerial parts			[73]
**14**	1-caffeoyl-β-d-glucose	Y/	*X. prockia*	Minas Gerais, Brazil	Leaves	Antifungal		[52]
**15**	8-hydroxy-6-methoxy-3-pentylisocoumarin	Y/Y	*X. longifolia*	Cuc Phuong, Vietnam	Stem bark	Antituberculosis	MIC: 40.5 µg/mL	[41]
**16**	Atraric acid	Y/Y	*X. longifolia* *X. velutina*	Manipur, India NS	Leaves Bark	Antifungal Antiproliferative		[40] [70]
**17**	Catechin	Y/Y	*X. longifolia* *X. controversa*	Manipur, India China	Leaves	Antifungal PDE inhibitor		[40] [65]
**18**	Genkwanin	Y/Y	*X. velutina*	Colombia	leaves, twigs and inflorescences	Immunomodulator		[72] [74]
**19**	Kaempferol	Y/Y	*X. longifolia*	Dehradun, India	Leaves	Antiproliferative		[75] [76]
**20**	Kaempferol-3-rhamnoside	Y/Y	*X. longifolia*	Dehradun, India	Leaves	Antioxidant		[75]
**21**	Kaempferol-3-β-xylopyranoside-4′-α-rhamnoside	Y/Y	*X. longifolia*	Dehradun, India	Leaves	Antioxidant		[75]
**22**	Quercetin	Y/Y	*X. longifolia*	Dehradun, India	Leaves	Antioxidant		[75] [77]
**23**	Quercetrin-3-rhamnoside	Y/Y	*X. longifolia*	Dehradun, India	Leaves	Antioxidant		[75]
**23**	Rutin	Y/	*X. longifolia*	Manipur, India	Leaves	Antifungal Antioxidant		[40]
**25**	Velutin	Y/Y	*X. velutina*	Colombia	Leaves, twigs and inflorescences			[72]
**26**	β-sitosterol	Y/Y	*X. longifolia*	Delhi, India	Leaves	Benign prostate hyperplasia symptom relief		[78] [79]
**27**	Lupeol	Y/Y	*X.flexuosa*	Guerrero, Mexico	Leaves	Anti-inflammatory		[80] [81]
**28**	Ugandensidial	Y/Y	*X. ciliatifolia*	Curitiba, Brazil	Root bark	Antibacterial *S. aureus S. epidermis*	MIC 62.5µg/mL	[30]
**29**	Friedelin	Y/Y	*X. controversa*	Guangxi, China	Stems	Antioxidant Hepatoprotective		[82] [83]
**30**	Velutinic acid	Y/Y	*X. velutina*	Colombia	leaves, twigs and inflorescences			[72]
**31**	*n*-hentriacontane	Y/Y	*X. longifolia*	Delhi, India	Leaves			[78]
**32**	Chaulmoogric acid	Y/Y	*X. controversa*	Guangxi, China	Stems	Antibacterial (leprosy) Neuroprotective		[82] [84] [85] [86]
**33**	2,6-dimethoxybenzoquinone	Y/Y	*X. velutina*	Colombia	Stem bark	Antibacterial Cytotoxic		[67]
**34**	(−) Syringaresinol	Y/Y	*X. controversa*	Guangxi, China	Stems		Bacteriostatic (*H. pylori*)	[82] [87]

Y: Yes; NS: Not Specified; PDE: phosphodiesterase; MIC: Minimum Inhibitory Concentration.

## References

[1] Hardy K. (2021). Paleomedicine and the Evolutionary Context of Medicinal Plant Use. Rev. Bras. Farmacogn..

[2] Domínguez-Martín E.M., Tavares J., Ríjo P., Díaz-Lanza A.M. (2020). Zoopharmacology: A Way to Discover New Cancer Treatments. Biomolecules.

[3] Solecki R.S. (1975). Shanidar IV, a Neanderthal Flower Burial in Northern Iraq. Science.

[4] ur Rehman F., Kalsoom M., Adnan M., Fazeli-Nasab B., Naz N., Ilahi H., Ali M.F., Ilyas M.A., Yousaf G., Toor M.D. (2021). Importance of Medicinal Plants in Human and Plant Pathology: A Review. Int. J. Pharm. Biomed. Rese.

[5] Marrelli M. (2021). Medicinal Plants. Plants.

[6] Jamshidi-Kia F., Lorigooini Z., Amini-Khoei H. (2018). Medicinal Plants: Past History and Future Perspective. J. Herbmed Pharmacol..

[7] Katiyar C., Kanjilal S., Gupta A., Katiyar S. (2012). Drug Discovery from Plant Sources: An Integrated Approach. AYU Int. Q. J. Res. Ayurveda.

[8] Calixto J.B. (2019). The Role of Natural Products in Modern Drug Discovery. An. Acad. Bras. Ciênc..

[9] *Salicaceae* Mirb.|Plants of the World Online|Kew Science. http://powo.science.kew.org/taxon/urn:lsid:ipni.org:names:30002644-2.

[10] *Xylosma* G. Forst.|Plants of the World Online|Kew Science. http://powo.science.kew.org/taxon/urn:lsid:ipni.org:names:332071-2.

[11] WFO *Xylosma* G. Forst. http://www.worldfloraonline.org/taxon/wfo-4000041044;jsessionid=402EBB0471E61A8E4D367F550003835E.

[12] The Angiosperm Phylogeny Group (2016). An Update of the Angiosperm Phylogeny Group Classification for the Orders and Families of Flowering Plants: APG IV. Bot. J. Linn. Soc..

[13] Uphof J. (1968). The Dictionary of Economic Plants.

[14] Berry S.S., Chapman J., Jones G., Jones J., Pass J., Roper C.F.E., Wilkes J. (1810). Encyclopaedia Londinensis, or, Universal Dictionary of Arts, Sciences, and Literature: Comprehending, under One General Alphabetical Arrangement, All the Words and Substance of Every Kind of Dictionary Extant in the English Language: In Which the Improved Departments of the Mechanical Compiled, Digested, and Arranged, by John Wilkes, of Milland House…; Assisted by Eminent Scholars of the English, Scotch, and Irish, Universities.

[15] Woodson R.E., Schery R.W., Robyns A. (1968). Flora of Panama. Family 128. Flacourtiaceae. Ann. Mo. Bot. Gard..

[16] Vossler F.G. (2012). Flower Visits, Nesting and Nest Defence Behaviour of Stingless Bees (*Apidae*: *Meliponini*): Suitability of the Bee Species for Meliponiculture in the Argentinean Chaco Region. Apidologie.

[17] García R., Pegüero B., Jiménez F., Veloz A., Clase T. (2016). Lista Roja de La Flora Vascular en República Dominicana.

[18] Reeves R.D., Baker A.J.M., Jaffré T., Erskine P.D., Echevarria G., Ent A. (2018). A Global Database for Plants That Hyperaccumulate Metal and Metalloid Trace Elements. New Phytol..

[19] Seregin I.V., Kozhevnikova A.D. (2006). Physiological Role of Nickel and Its Toxic Effects on Higher Plants. Russ. J. Plant Physiol..

[20] Chaney R.L., Angle J.S., Broadhurst C.L., Peters C.A., Tappero R.V., Sparks D.L. (2007). Improved Understanding of Hyperaccumulation Yields Commercial Phytoextraction and Phytomining Technologies. J. Environ. Qual..

[21] IUCN The IUCN Red List of Threatened Species. https://www.iucnredlist.org/en.

[22] Digital Science Dimensions [Software]. https://app.dimensions.ai/analytics/publication/overview/timeline.

[23] Harzing A.W. (2021). Publish or Perish.

[24] Zhang Z.-S., Zeng Q.-Y., Liu Y.-J. (2021). Frequent Ploidy Changes in Salicaceae Indicates Widespread Sharing of the Salicoid Whole Genome Duplication by the Relatives of *Populus* L. and *Salix* L.. BMC Plant Biol..

[25] Ye H., Li C., Ye W., Zeng F., Liu F., Liu Y., Wang F., Ye Y., Fu L., Li J., Ye H., Li C., Ye W., Zeng F. (2021). Medicinal Angiosperms of *Flacourtiaceae*, *Tamaricaceae*, *Passifloraceae*, and *Cucurbitaceae*. Common Chinese Materia Medica.

[26] WHO Anatomical Therapeutic Chemical (ATC) Classification. https://www.who.int/tools/atc-ddd-toolkit/atc-classification.

[27] Van den Berg M.E., da Silva M.H.L., da Silva M.G. Plantas Aromáticas Da Amazônia. Proceedings of the 1er Simpósio do Trópico Úmido.

[28] Rodríguez-Flores O., Torréz-Centeno E., Valenzuela-Betanco R. (2005). Plantas Utilizadas Para el Tratamiento de Enfermedades en los Animales Domésticos, Reserva Natural El Tisey, Estelí. Ph.D. Thesis.

[29] Juep A. (2008). Rescate del Conocimiento Tradicional y Biológico Para el Manejo de Productos Forestales no Maderables en la Comunidad Indígena Jameykari, Costa Rica. Master’s Thesis.

[30] Philippsen A.F., Miguel O.G., Miguel M.D., de Lima C.P., Kalegari M., Lordello A.L.L. (2013). Validation of the antibacterial activity of root bark of *Xylosma ciliatifolia* (Clos) Eichler (*Flacourtiaceae*/*Salicaceae sensu lato*). Rev. Cuba. Plantas Med..

[31] Shizhen L. (2021). Ben Cao Gang Mu, Volume II: Waters, Fires, Soils, Metals, Jades, Stones, Minerals, Salts.

[32] Lee J.Y., Ahn E.-K., Ko H.-J., Cho Y.-R., Ko W.C., Jung Y.-H., Choi K.-M., Choi M.-R., Oh J.S. (2014). Anti-Melanogenic, Anti-Wrinkle, Anti-Inflammatory and Anti-Oxidant Effects of *Xylosma congesta* Leaf Ethanol Extract. J. Appl. Biol. Chem..

[33] Yungui W. (2018). A Disease Control Method for Suckling Piglets. Patent.

[34] Duke J.A., Ayensu E.S. (1985). Medicinal Plants of China.

[35] Xu Z.-R., Chai X.-Y., Bai C.-C., Ren H.-Y., Lu Y.-N., Shi H.-M., Tu P.-F. (2008). Xylocosides A-G, Phenolic Glucosides from the Stems of *Xylosma controversum*. Helv. Chim. Acta.

[36] Cornejo-Báez A. (2016). Evaluación de la actividad antibacteriana de los extractos y fracciones de *Bidens pilosa* L. y *Xylosma flexuosum* (H. B. & K.) Hemsl y estudio quimiométrico de la actividad antituberculosa de los perfiles cromatográficos de *Bidens pilosa* L.. Master’s Thesis.

[37] Grijalva Pineda A., Ministerio del Ambiente y Recursos Naturales de Nicaragua (2006). Flora útil: Etnobotánica de Nicaragua.

[38] Pérez-Torres F. (2007). Manual de Plantas Medicinales más Comunes del Occidente de Nicaragua.

[39] Grandtner M.M. (2005). Elsevier’s Dictionary of Trees: With Names in Latin, English, French, Spanish and Other Languages.

[40] Devi W.R., Singh S.B., Singh C.B. (2013). Antioxidant and Anti-Dermatophytic Properties Leaf and Stem Bark of *Xylosma longifolium* Clos. BMC Complement. Altern. Med..

[41] Truong B.N., Pham V.C., Mai H.D.T., Nguyen V.H., Nguyen M.C., Nguyen T.H., Zhang H., Fong H.H.S., Franzblau S.G., Soejarto D.D. (2011). Chemical Constituents from *Xylosma longifolia* and Their Anti-Tubercular Activity. Phytochem. Lett..

[42] Swapana N., Noji M., Nishiuma R., Izumi M., Imagawa H., Kasai Y., Okamoto Y., Iseki K., Singh C.B., Asakawa Y. (2017). A New Diphenyl Ether Glycoside from *Xylosma longifolium* Collected from North-East India. Nat. Prod. Commun..

[43] Salam S. (2020). Medicinal Plant Used for the Treatment of Muscular Sprain by the Tangkhul Tribe of Ukhrul District, Manipur, India. Int. J. Adv. Res..

[44] Khare C.P. (2007). Indian Medicinal Plants: An Illustrated Dictionary.

[45] Joly L.G., Guerra S., Séptimo R., Solís P.N., Correa M., Gupta M., Levy S., Sandberg F. (1987). Ethnobotanical Inventory of Medicinal Plants Used by the Guaymi Indians in Western Panama. Part I. J. Ethnopharmacol..

[46] García-Barriga H. (1992). Flora Medicinal de Colombia: Botánica Médica.

[47] Cerón C., Montalvo C. (2000). Reserva Biológica Limoncocha: Formaciones Vegetales, Diversidad y Etnobotánica. Cinchonia.

[48] Caballero-George C., Gupta M. (2011). A Quarter Century of Pharmacognostic Research on Panamanian Flora: A Review. Planta Med..

[49] Brown M. (1984). Una paz Incierta: Comunidades Aguarunas Frente al Impacto de la Carretera Marginal.

[50] Vasquez Y. (2016). Biological and Chemical Investigation of Panamanian Plants for Potential Utility against Metabolic Syndrome.

[51] Setzer M.C., Moriarity D.M., Lawton R.O., Setzer W.N., Gentry G.A., Haber W.A. (2003). Phytomedicinal Potential of Tropical Cloudforest Plants from Monteverde, Costa Rica. Rev. Biol. Trop..

[52] Folly M.L.C., Ferreira G.F., Salvador M.R., Sathler A.A., da Silva G.F., Santos J.C.B., dos Santos J.R.A., Nunes Neto W.R., Rodrigues J.F.S., Fernandes E.S. (2020). Evaluation of In Vitro Antifungal Activity of *Xylosma prockia* (Turcz.) Turcz. (*Salicaceae*) Leaves Against *Cryptococcus* spp.. Front. Microbiol..

[53] Mosaddik M.A., Banbury L., Forster P., Booth R., Markham J., Leach D., Waterman P.G. (2004). Screening of Some Australian *Flacourtiaceae* Species for in vitro Antioxidant, Cytotoxic and Antimicrobial Activity. Phytomedicine.

[54] Castro S.B.R., Leal C.A.G., Freire F.R., Carvalho D.A., Oliveira D.F., Figueiredo H.C.P. (2008). Antibacterial Activity of Plant Extracts from Brazil against Fish Pathogenic Bacteria. Braz. J. Microbiol..

[55] Kuete V., Seo E.-J., Krusche B., Oswald M., Wiench B., Schröder S., Greten H.J., Lee I.-S., Efferth T. (2013). Cytotoxicity and Pharmacogenomics of Medicinal Plants from Traditional Korean Medicine. Evid. Based Complement. Alternat. Med..

[56] Jones E., Ekundayo O., Kingston D.G.I. (1981). Plant Anticancer Agents. XI. 2,6-Dimethoxybenzoquinone as a Cytotoxic Constituent of *Tibouchina pulchra*. J. Nat. Prod..

[57] Altemimi A., Lakhssassi N., Baharlouei A., Watson D., Lightfoot D. (2017). Phytochemicals: Extraction, Isolation, and Identification of Bioactive Compounds from Plant Extracts. Plants.

[58] Nassar M., Sáenz J.A., Galvez N. (1980). Phytochemical Screening of Costa Rican Plants: Alkaloid Analysis. V. Rev. Biol. Trop..

[59] Bhattacharyya R., Boruah J., Medhi K., Borkataki S. (2020). Phytochemical Analysis of Leaves of *Xylosma longifolia* Clos.: A Plant of Ethnomedicinal Importance. Int. J. Pharm. Sci. Res..

[60] Bhaumik P.K., Guha K.P., Biswas G.K., Mukherjee B. (1987). (−)Flacourtin, a Phenolic Glucoside Ester from *Flacourtia indica*. Phytochemistry.

[61] Sashidhara K.V., Singh S.P., Singh S.V., Srivastava R.K., Srivastava K., Saxena J.K., Puri S.K. (2013). Isolation and Identification of β-Hematin Inhibitors from *Flacourtia indica* as Promising Antiplasmodial Agents. Eur. J. Med. Chem..

[62] Bourjot M., Leyssen P., Eydoux C., Guillemot J.-C., Canard B., Rasoanaivo P., Guéritte F., Litaudon M. (2012). Flacourtosides A–F, Phenolic Glycosides Isolated from *Flacourtia ramontchi*. J. Nat. Prod..

[63] Prangé T., Lavaud C., Massiot G. (2021). A Reappraisal of the Structure of Xylosmin. Phytochem. Lett..

[64] Ghavre M., Froese J., Murphy B., Simionescu R., Hudlicky T. (2017). A Formal Approach to Xylosmin and Flacourtosides E and F: Chemoenzymatic Total Synthesis of the Hydroxylated Cyclohexenone Carboxylic Acid Moiety of Xylosmin. Org. Lett..

[65] Xiao-Ping P. (2010). Inhibition of Phosphodiesterase Activity by Chemical Constituents of *Xylosma controversum* Clos and Scolopia Chinensis(Lour.) Clos. Chin. J. New Drugs.

[66] Yu Y., Zhou L., Sun M., Zhou T., Zhong K., Wang H., Liu Y., Liu X., Xiao R., Ge J. (2012). Xylocoside G Reduces Amyloid-β Induced Neurotoxicity by Inhibiting NF-ΚB Signaling Pathway in Neuronal Cells. J. Alzheimers Dis..

[67] Cordell G.A., Chang P.T., Fong H.H.S., Farnsworth. (1977). Xylosmacin, a New Phenolic Glucoside Ester from *Xylosma velutina* (Flacourtiaceae). Lloydia-J. Nat. Prod..

[68] Wang X., Yu W., Lou H. (2005). Antifungal Constituents from the Chinese Moss *Homalia trichomanoides*. Chem. Biodivers..

[69] Mun S.-K., Kang K.-Y., Jang H.-Y., Hwang Y.-H., Hong S.-G., Kim S.-J., Cho H.-W., Chang D.-J., Hur J.-S., Yee S.-T. (2020). Atraric Acid Exhibits Anti-Inflammatory Effect in Lipopolysaccharide-Stimulated RAW264.7 Cells and Mouse Models. Int. J. Mol. Sci..

[70] Papaioannou M., Schleich S., Prade I., Degen S., Roell D., Schubert U., Tanner T., Claessens F., Matusch R., Baniahmad A. (2009). The Natural Compound Atraric Acid Is an Antagonist of the Human Androgen Receptor Inhibiting Cellular Invasiveness and Prostate Cancer Cell Growth. J. Cell. Mol. Med..

[71] Hoffmann H.-R., Matusch R., Baniahmad A. (2013). Isolation of Atraric Acid, Synthesis of Atraric Acid Derivatives, and Use of Atraric Acid and the Derivatives Thereof for the Treatment of Benign Prostatic Hyperplasia, Prostate Carcinoma and Spinobulbar Muscular Atrophy. U.S. Patent.

[72] Chang P.T.O., Cordell G.A., Fong H.H.S., Farnsworth N.R. (1977). Velutinic Acid, a New Friedelane Derivative from *Xylosma velutina* (*Flacourtiaceae*). Phytochemistry.

[73] Gibbons S., Gray A.I., Waterman P.G., Hockless D.C.R., Skelton B.W., White A.H. (1995). Benzoylated Derivatives of 2-β-Glucopyranosyloxy-2,5-Dihydroxybenzyl Alcohol from *Xylosma flexuosum*: Structure and Relative Configuration of Xylosmin. J. Nat. Prod..

[74] Nasr-Bouzaiene N., Sassi A., Bedoui A., Krifa M., Chekir-Ghedira L., Ghedira K. (2016). Immunomodulatory and Cellular Antioxidant Activities of Pure Compounds from *Teucrium ramosissimum* Desf. Tumor Biol..

[75] Parveen M., Ghalib R.M. (2012). Flavonoids and Antimicrobial Activity of Leaves of *Xylosma longifolium*. J. Chil. Chem. Soc..

[76] Zhang Y., Chen A.Y., Li M., Chen C., Yao Q. (2008). *Ginkgo biloba* Extract Kaempferol Inhibits Cell Proliferation and Induces Apoptosis in Pancreatic Cancer Cells. J. Surg. Res..

[77] Xu D., Hu M.-J., Wang Y.-Q., Cui Y.-L. (2019). Antioxidant Activities of Quercetin and Its Complexes for Medicinal Application. Molecules.

[78] Sultana S., Ali M., Jameel M. (2018). Aliphatic Constituents from the Leaves of *Dillenia indica* L., *Halothamus bottae* Jaub. and *Xylosma longifolium* Clos. Chem. Res. J..

[79] Wilt T.J., Ishani A., MacDonald R., Stark G., Mulrow C.D., Lau J. (1999). Beta-Sitosterols for Benign Prostatic Hyperplasia. Cochrane Database Syst. Rev..

[80] Acton P., Ashton Q. (2012). Advances in Chlorophyll Research and Application.

[81] Liu K., Zhang X., Xie L., Deng M., Chen H., Song J., Long J., Li X., Luo J. (2021). Lupeol and Its Derivatives as Anticancer and Anti-Inflammatory Agents: Molecular Mechanisms and Therapeutic Efficacy. Pharmacol. Res..

[82] Hong-Yan R., Ya-Nan L., Xing-Yun C., Peng-Fei T., Zheng-Ren X. (2007). Chemical Constituents from *Xylosma controversum*. J. Chin. Pharm. Sci..

[83] Sunil C., Duraipandiyan V., Ignacimuthu S., Al-Dhabi N.A. (2013). Antioxidant, Free Radical Scavenging and Liver Protective Effects of Friedelin Isolated from *Azima tetracantha* Lam. Leaves. Food Chem..

[84] Levy L. (1975). The Activity of Chaulmoogra Acids against *Mycobacterium leprae*. Am. Rev. Respir. Dis..

[85] Cabot M.C., Goucher C.R. (1981). Chaulmoogric Acid: Assimilation into the Complex Lipids of Mycobacteria. Lipids.

[86] Cher C., Tremblay M.-H., Barber J.R., Chung Ng S., Zhang B. (2010). Identification of Chaulmoogric Acid as a Small Molecule Activator of Protein Phosphatase 5. Appl. Biochem. Biotechnol..

[87] Miyazawa M., Utsunomiya H., Inada K., Yamada T., Okuno Y., Tanaka H., Tatematsu M. (2006). Inhibition of *Helicobacter pylori* Motility by (+)-Syringaresinol from Unripe Japanese Apricot. Biol. Pharm. Bull..

